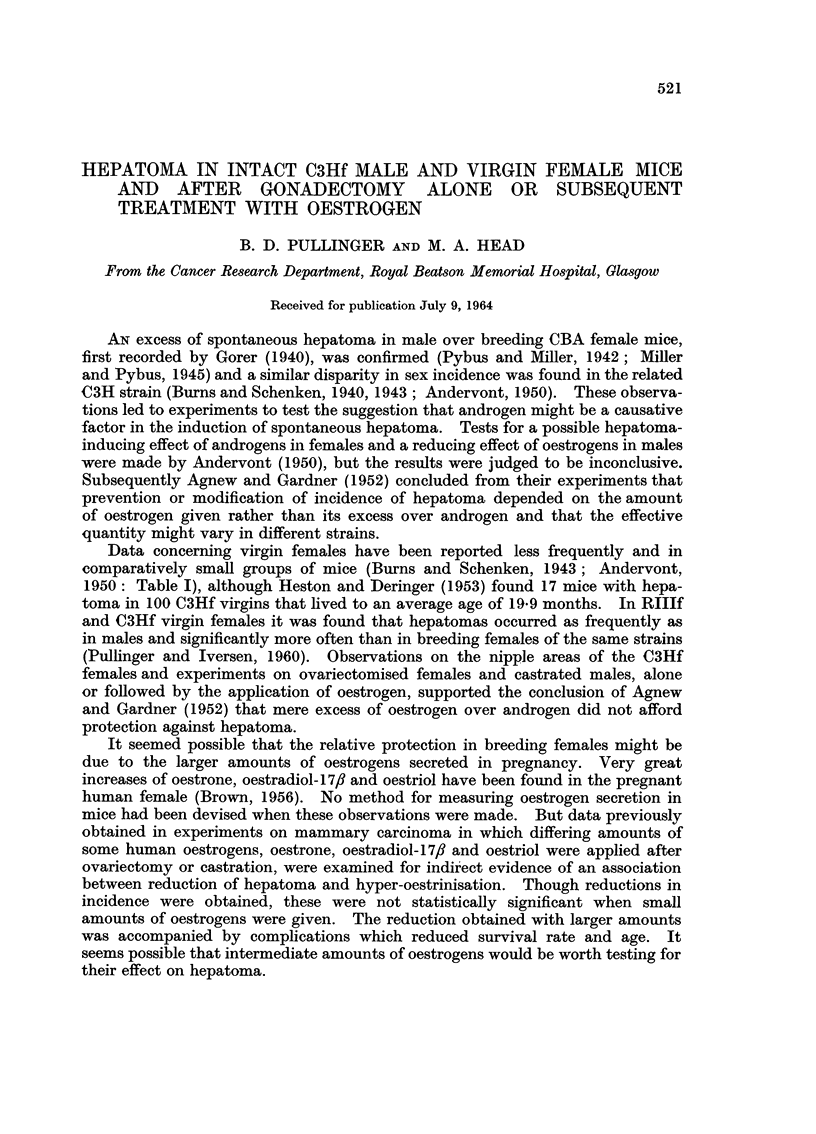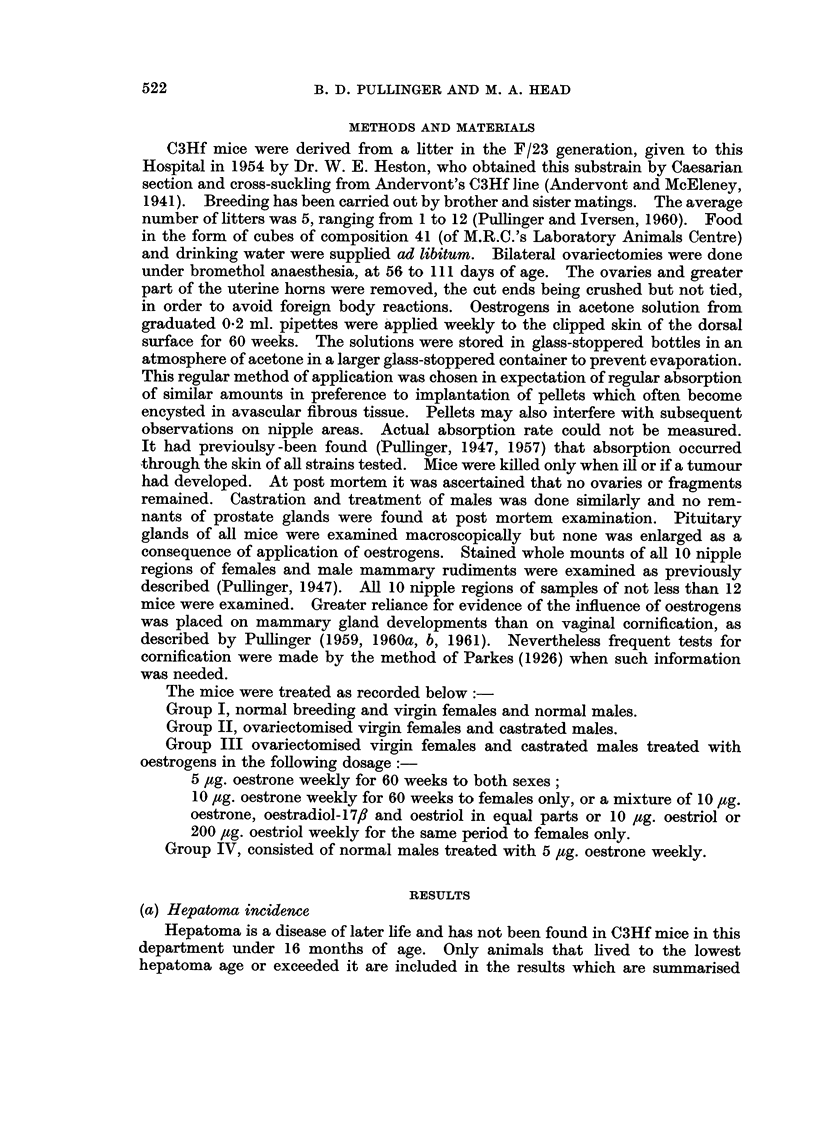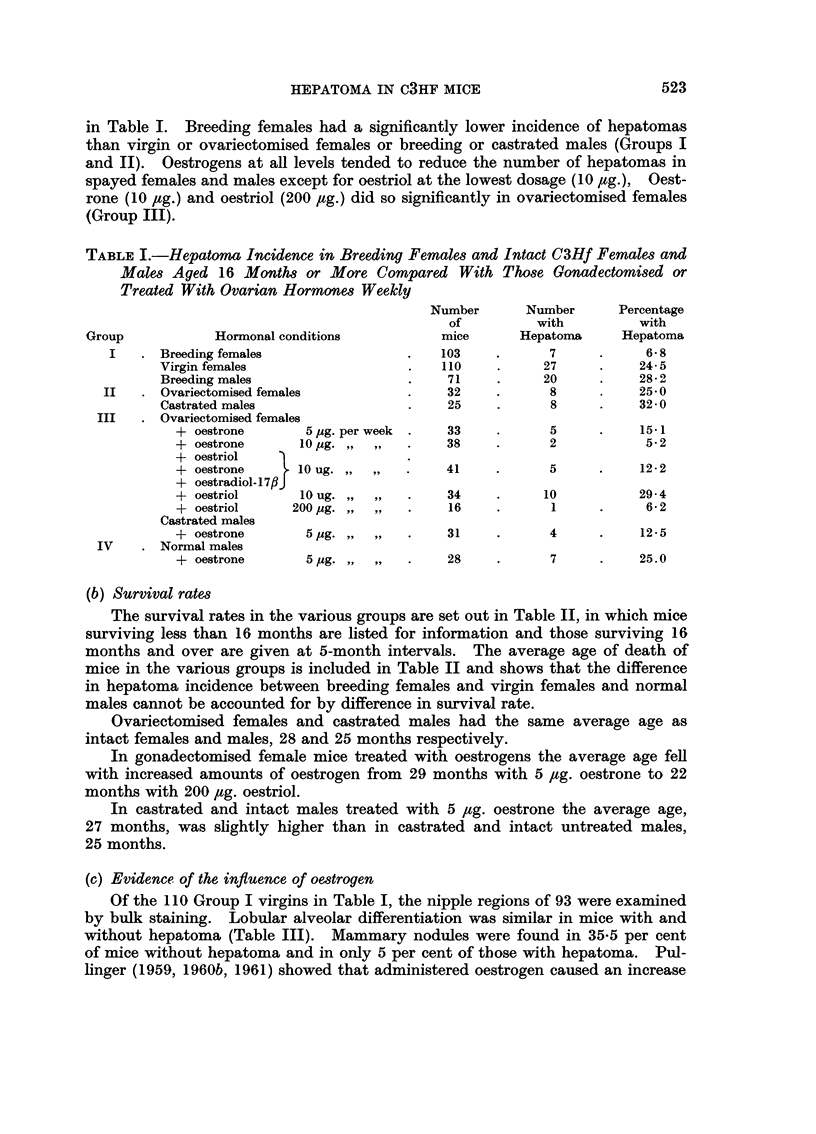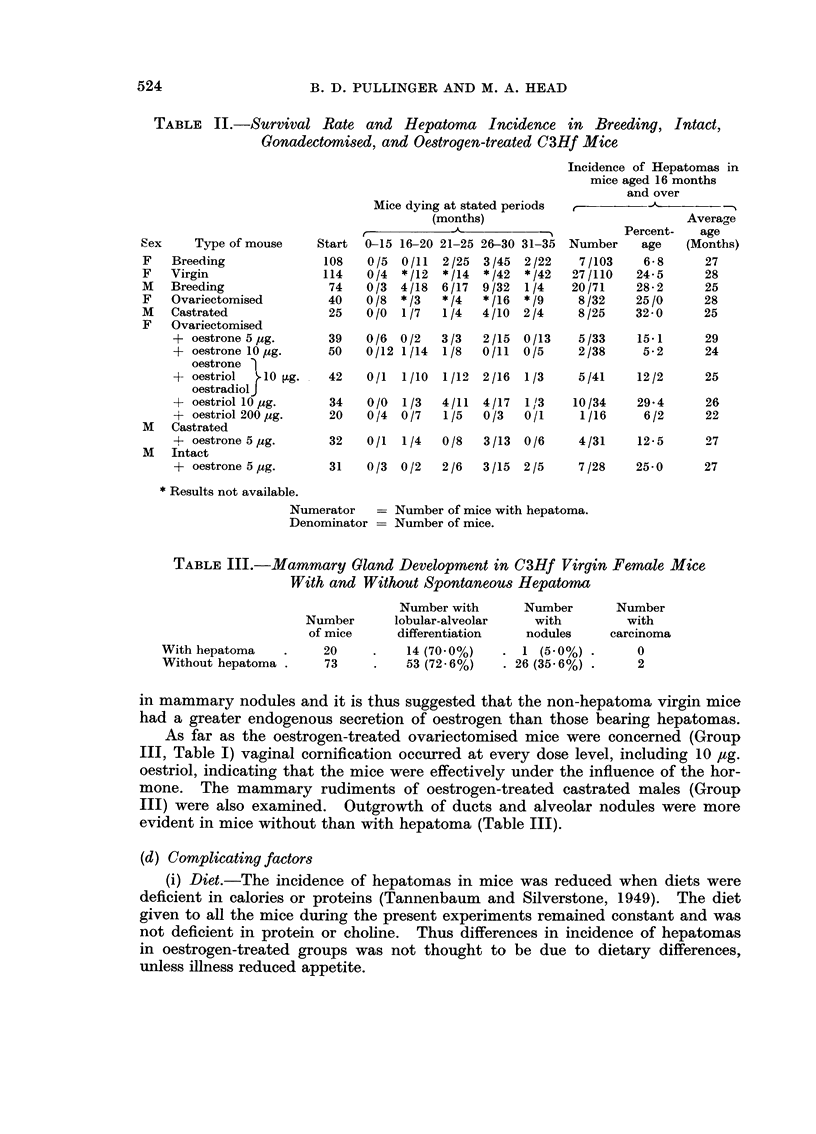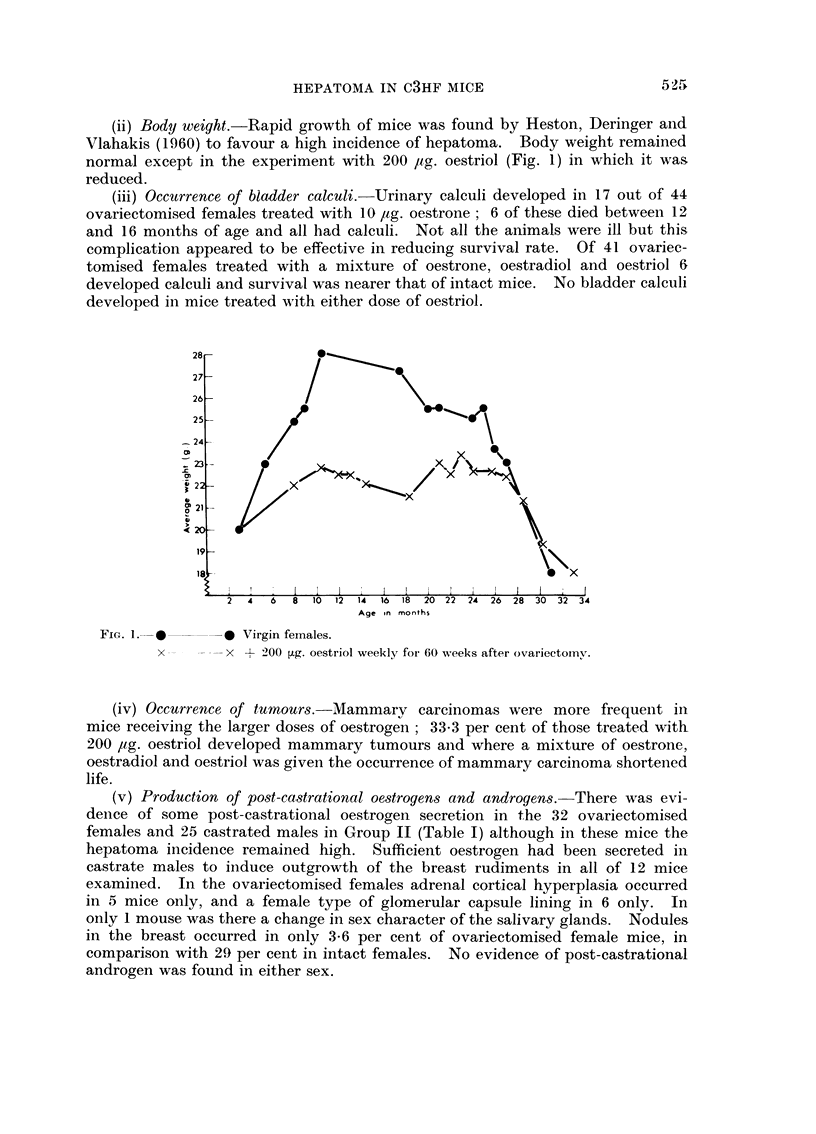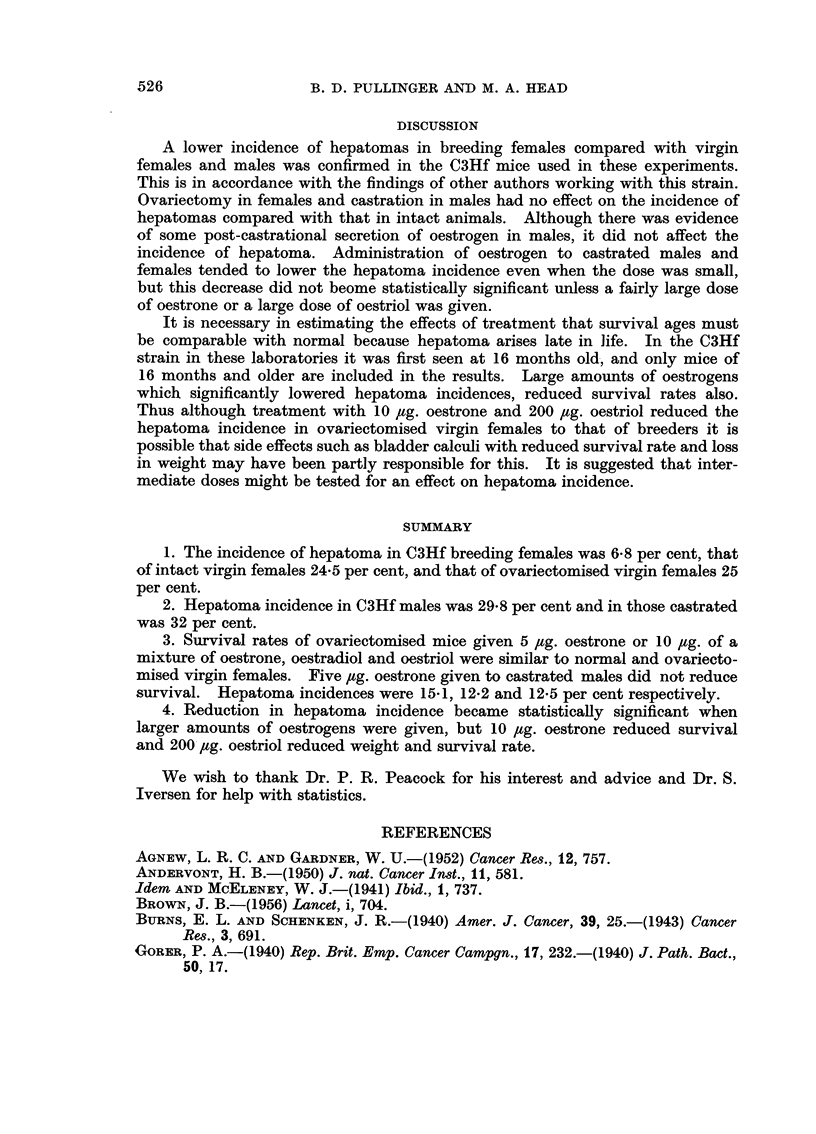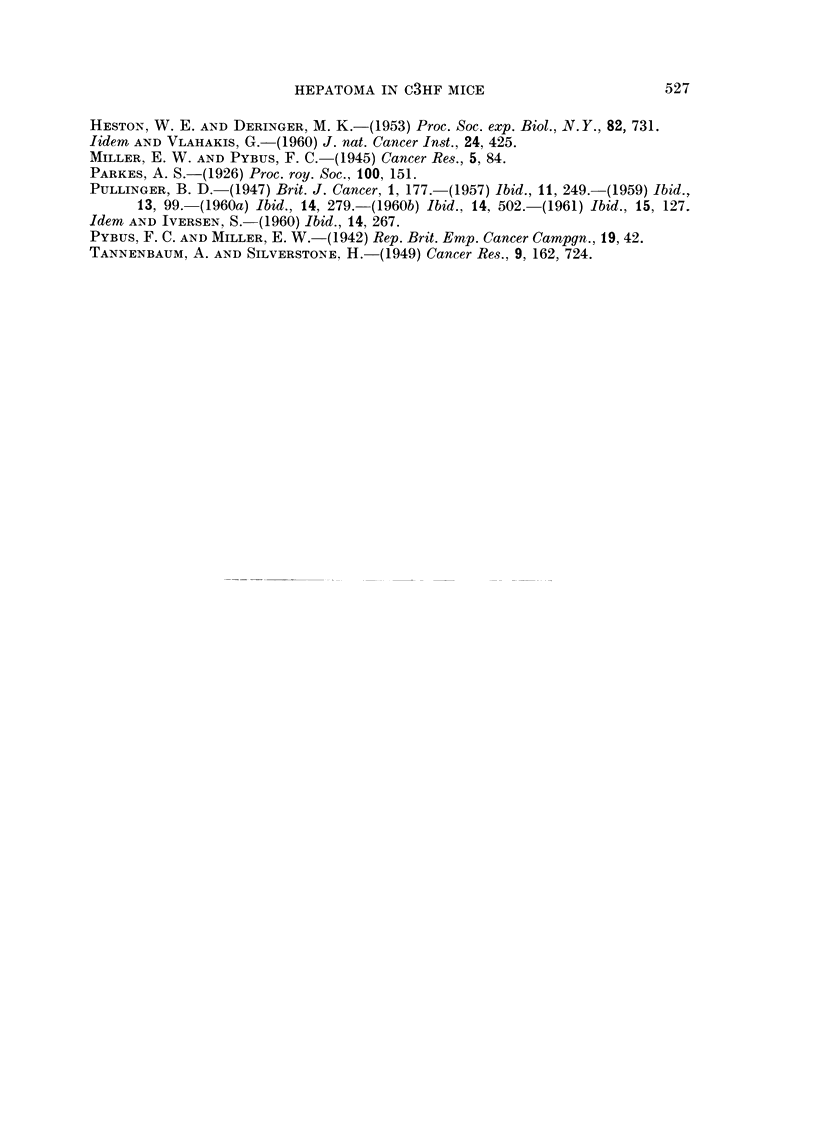# Hepatoma in Intact C3Hf Male and Virgin Female Mice and after Gonadectomy alone or subsequent Treatment with Oestrogen

**DOI:** 10.1038/bjc.1964.59

**Published:** 1964-09

**Authors:** B. D. Pullinger, M. A. Head


### HEPATOMA IN INTACT C3Hf MALE AND VIRGIN FEMALE MICE AND AFTER GONADECTOMY ALONE OR SUBSEQUENT TREATMENT WITH OESTROGEN

B. D. PULLINGER AND M. A. HEAD

*From the Cancer Research Department, Royal Beatson Memorial Hospital, Glasgow*

Received for publication July 9, 1964

AN excess of spontaneous hepatoma in male over breeding CBA female mice, first recorded by Gorer (1940), was confirmed (Pybus and Miller, 1942 ; Miller and Pybus, 1945) and a similar disparity in sex incidence was found in the related C3H strain (Burns and Schenken, 1940, 1943 ; Andervont, 1950). These observations led to experiments to test the suggestion that androgen might be a causative factor in the induction of spontaneous hepatoma. Tests for a possible hepatoma-inducing effect of androgens in females and a reducing effect of oestrogens in males were made by Andervont (1950), but the results were judged to be inconclusive. Subsequently Agnew and Gardner (1952) concluded from their experiments that prevention or modification of incidence of hepatoma depended on the amount of oestrogen given rather than its excess over androgen and that the effective quantity might vary in different strains.

Data concerning virgin females have been reported less frequently and in comparatively small groups of mice (Burns and Schenken, 1943 ; Andervont, 1950 : Table I), although Heston and Deringer (1953) found 17 mice with hepatoma in 100 C3Hf virgins that lived to an average age of 19·9 months. In RIIIf and C3Hf virgin females it was found that hepatomas occurred as frequently as in males and significantly more often than in breeding females of the same strains (Pullinger and Iversen, 1960). Observations on the nipple areas of the C3Hf females and experiments on ovariectomised females and castrated males, alone or followed by the application of oestrogen, supported the conclusion of Agnew and Gardner (1952) that mere excess of oestrogen over androgen did not afford protection against hepatoma.

It seemed possible that the relative protection in breeding females might be due to the larger amounts of oestrogens secreted in pregnancy. Very great increases of oestrone, oestradiol-17$\beta$ and oestriol have been found in the pregnant human female (Brown, 1956). No method for measuring oestrogen secretion in mice had been devised when these observations were made. But data previously obtained in experiments on mammary carcinoma in which differing amounts of some human oestrogens, oestrone, oestradiol-17$\beta$ and oestriol were applied after ovariectomy or castration, were examined for indirect evidence of an association between reduction of hepatoma and hyper-oestrinisation. Though reductions in incidence were obtained, these were not statistically significant when small amounts of oestrogens were given. The reduction obtained with larger amounts was accompanied by complications which reduced survival rate and age. It seems possible that intermediate amounts of oestrogens would be worth testing for their effect on hepatoma.

### METHODS AND MATERIALS

C3Hf mice were derived from a litter in the F/23 generation, given to this Hospital in 1954 by Dr. W. E. Heston, who obtained this substrain by Caesarian section and cross-suckling from Andervont's C3Hf line (Andervont and McEleney, 1941). Breeding has been carried out by brother and sister matings. The average number of litters was 5, ranging from 1 to 12 (Pullinger and Iversen, 1960). Food in the form of cubes of composition 41 (of M.R.C.'s Laboratory Animals Centre) and drinking water were supplied *ad libitum*. Bilateral ovariectomies were done under bromethol anaesthesia, at 56 to 111 days of age. The ovaries and greater part of the uterine horns were removed, the cut ends being crushed but not tied, in order to avoid foreign body reactions. Oestrogens in acetone solution from graduated 0·2 ml. pipettes were applied weekly to the clipped skin of the dorsal surface for 60 weeks. The solutions were stored in glass-stoppered bottles in an atmosphere of acetone in a larger glass-stoppered container to prevent evaporation. This regular method of application was chosen in expectation of regular absorption of similar amounts in preference to implantation of pellets which often become encysted in avascular fibrous tissue. Pellets may also interfere with subsequent observations on nipple areas. Actual absorption rate could not be measured. It had previoulsy-been found (Pullinger, 1947, 1957) that absorption occurred through the skin of all strains tested. Mice were killed only when ill or if a tumour had developed. At post mortem it was ascertained that no ovaries or fragments remained. Castration and treatment of males was done similarly and no remnants of prostate glands were found at post mortem examination. Pituitary glands of all mice were examined macroscopically but none was enlarged as a consequence of application of oestrogens. Stained whole mounts of all 10 nipple regions of females and male mammary rudiments were examined as previously described (Pullinger, 1947). All 10 nipple regions of samples of not less than 12 mice were examined. Greater reliance for evidence of the influence of oestrogens was placed on mammary gland developments than on vaginal cornification, as described by Pullinger (1959, 1960*a*, *b*, 1961). Nevertheless frequent tests for cornification were made by the method of Parkes (1926) when such information was needed.

The mice were treated as recorded below :—

Group I, normal breeding and virgin females and normal males.

Group II, ovariectomised virgin females and castrated males.

Group III ovariectomised virgin females and castrated males treated with oestrogens in the following dosage :—

5 $\mu$g. oestrone weekly for 60 weeks to both sexes ;

10 $\mu$g. oestrone weekly for 60 weeks to females only, or a mixture of 10 $\mu$g. oestrone, oestradiol-17$\beta$ and oestriol in equal parts or 10 $\mu$g. oestriol or 200 $\mu$g. oestriol weekly for the same period to females only.

Group IV, consisted of normal males treated with 5 $\mu$g. oestrone weekly.

### RESULTS

### (*a*) *Hepatoma incidence*

Hepatoma is a disease of later life and has not been found in C3Hf mice in this department under 16 months of age. Only animals that lived to the lowest hepatoma age or exceeded it are included in the results which are summarised in Table I. Breeding females had a significantly lower incidence of hepatomas than virgin or ovariectomised females or breeding or castrated males (Groups I and II). Oestrogens at all levels tended to reduce the number of hepatomas in spayed females and males except for oestriol at the lowest dosage (10 $\mu$g.), Oestrone (10 $\mu$g.) and oestriol (200 $\mu$g.) did so significantly in ovariectomised females (Group III).

TABLE I.—*Hepatoma Incidence in Breeding Females and Intact C3Hf Females and Males Aged* 16 *Months or More Compared With Those Gonadectomised or Treated With Ovarian Hormones Weekly*

| Group | Hormonal conditions | | Number of mice | Number with Hepatoma | Percentage with Hepatoma |
|---|---|---|---|---|---|
| I | Breeding females | | 103 | 7 | 6·8 |
| | Virgin females | | 110 | 27 | 24·5 |
| | Breeding males | | 71 | 20 | 28·2 |
| II | Ovariectomised females | | 32 | 8 | 25·0 |
| | Castrated males | | 25 | 8 | 32·0 |
| III | Ovariectomised females | | | | |
| | + oestrone | 5 $\mu$g. per week | 33 | 5 | 15·1 |
| | + oestrone | 10 $\mu$g. ,, ,, | 38 | 2 | 5·2 |
| | + oestriol<br>+ oestrone<br>+ oestradiol-17$\beta$ | 10 ug. ,, ,, | 41 | 5 | 12·2 |
| | + oestriol | 10 ug. ,, ,, | 34 | 10 | 29·4 |
| | + oestriol | 200 $\mu$g. ,, ,, | 16 | 1 | 6·2 |
| | Castrated males | | | | |
| | + oestrone | 5 $\mu$g. ,, ,, | 31 | 4 | 12·5 |
| IV | Normal males | | | | |
| | + oestrone | 5 $\mu$g. ,, ,, | 28 | 7 | 25.0 |

(*b*) *Survival rates*

The survival rates in the various groups are set out in Table II, in which mice surviving less than 16 months are listed for information and those surviving 16 months and over are given at 5-month intervals. The average age of death of mice in the various groups is included in Table II and shows that the difference in hepatoma incidence between breeding females and virgin females and normal males cannot be accounted for by difference in survival rate.

Ovariectomised females and castrated males had the same average age as intact females and males, 28 and 25 months respectively.

In gonadectomised female mice treated with oestrogens the average age fell with increased amounts of oestrogen from 29 months with 5 $\mu$g. oestrone to 22 months with 200 $\mu$g. oestriol.

In castrated and intact males treated with 5 $\mu$g. oestrone the average age, 27 months, was slightly higher than in castrated and intact untreated males, 25 months.

(*c*) *Evidence of the influence of oestrogen*

Of the 110 Group I virgins in Table I, the nipple regions of 93 were examined by bulk staining. Lobular alveolar differentiation was similar in mice with and without hepatoma (Table III). Mammary nodules were found in 35·5 per cent of mice without hepatoma and in only 5 per cent of those with hepatoma. Pullinger (1959, 1960*b*, 1961) showed that administered oestrogen caused an increase

TABLE II.—*Survival Rate and Hepatoma Incidence in Breeding, Intact, Gonadectomised, and Oestrogen-treated C3Hf Mice*

| | | | Mice dying at stated periods (months) | | | | | Incidence of Hepatomas in mice aged 16 months and over | | |
|---|---|---|---|---|---|---|---|---|---|---|
| Sex | Type of mouse | Start | 0–15 | 16–20 | 21–25 | 26–30 | 31–35 | Number | Percentage | Average age (Months) |
| F | Breeding | 108 | 0/5 | 0/11 | 2/25 | 3/45 | 2/22 | 7/103 | 6·8 | 27 |
| F | Virgin | 114 | 0/4 | */12 | */14 | */42 | */42 | 27/110 | 24·5 | 28 |
| M | Breeding | 74 | 0/3 | 4/18 | 6/17 | 9/32 | 1/4 | 20/71 | 28·2 | 25 |
| F | Ovariectomised | 40 | 0/8 | */3 | */4 | */16 | */9 | 8/32 | 25/0 | 28 |
| M | Castrated | 25 | 0/0 | 1/7 | 1/4 | 4/10 | 2/4 | 8/25 | 32·0 | 25 |
| F | Ovariectomised | | | | | | | | | |
| | + oestrone 5 μg. | 39 | 0/6 | 0/2 | 3/3 | 2/15 | 0/13 | 5/33 | 15·1 | 29 |
| | + oestrone 10 μg. | 50 | 0/12 | 1/14 | 1/8 | 0/11 | 0/5 | 2/38 | 5·2 | 24 |
| | + oestrone, oestriol, oestradiol } 10 μg. | 42 | 0/1 | 1/10 | 1/12 | 2/16 | 1/3 | 5/41 | 12/2 | 25 |
| | + oestriol 10 μg. | 34 | 0/0 | 1/3 | 4/11 | 4/17 | 1/3 | 10/34 | 29·4 | 26 |
| | + oestriol 200 μg. | 20 | 0/4 | 0/7 | 1/5 | 0/3 | 0/1 | 1/16 | 6/2 | 22 |
| M | Castrated | | | | | | | | | |
| | + oestrone 5 μg. | 32 | 0/1 | 1/4 | 0/8 | 3/13 | 0/6 | 4/31 | 12·5 | 27 |
| M | Intact | | | | | | | | | |
| | + oestrone 5 μg. | 31 | 0/3 | 0/2 | 2/6 | 3/15 | 2/5 | 7/28 | 25·0 | 27 |

* Results not available.

Numerator = Number of mice with hepatoma.
Denominator = Number of mice.

TABLE III.—*Mammary Gland Development in C3Hf Virgin Female Mice With and Without Spontaneous Hepatoma*

| | Number of mice | Number with lobular-alveolar differentiation | Number with nodules | Number with carcinoma |
|---|---|---|---|---|
| With hepatoma | 20 | 14 (70·0%) | 1 (5·0%) | 0 |
| Without hepatoma | 73 | 53 (72·6%) | 26 (35·6%) | 2 |

in mammary nodules and it is thus suggested that the non-hepatoma virgin mice had a greater endogenous secretion of oestrogen than those bearing hepatomas.

As far as the oestrogen-treated ovariectomised mice were concerned (Group III, Table I) vaginal cornification occurred at every dose level, including 10 μg. oestriol, indicating that the mice were effectively under the influence of the hormone. The mammary rudiments of oestrogen-treated castrated males (Group III) were also examined. Outgrowth of ducts and alveolar nodules were more evident in mice without than with hepatoma (Table III).

### (*d*) *Complicating factors*

(i) *Diet.*—The incidence of hepatomas in mice was reduced when diets were deficient in calories or proteins (Tannenbaum and Silverstone, 1949). The diet given to all the mice during the present experiments remained constant and was not deficient in protein or choline. Thus differences in incidence of hepatomas in oestrogen-treated groups was not thought to be due to dietary differences, unless illness reduced appetite.

(ii) *Body weight.*—Rapid growth of mice was found by Heston, Deringer and Vlahakis (1960) to favour a high incidence of hepatoma. Body weight remained normal except in the experiment with 200 $\mu$g. oestriol (Fig. 1) in which it was reduced.

(iii) *Occurrence of bladder calculi.*—Urinary calculi developed in 17 out of 44 ovariectomised females treated with 10 $\mu$g. oestrone ; 6 of these died between 12 and 16 months of age and all had calculi. Not all the animals were ill but this complication appeared to be effective in reducing survival rate. Of 41 ovariectomised females treated with a mixture of oestrone, oestradiol and oestriol 6 developed calculi and survival was nearer that of intact mice. No bladder calculi developed in mice treated with either dose of oestriol.

FIG. 1.—●———● Virgin females.

×– – –× + 200 µg. oestriol weekly for 60 weeks after ovariectomy.

(iv) *Occurrence of tumours.*—Mammary carcinomas were more frequent in mice receiving the larger doses of oestrogen ; 33·3 per cent of those treated with 200 $\mu$g. oestriol developed mammary tumours and where a mixture of oestrone, oestradiol and oestriol was given the occurrence of mammary carcinoma shortened life.

(v) *Production of post-castrational oestrogens and androgens.*—There was evidence of some post-castrational oestrogen secretion in the 32 ovariectomised females and 25 castrated males in Group II (Table I) although in these mice the hepatoma incidence remained high. Sufficient oestrogen had been secreted in castrate males to induce outgrowth of the breast rudiments in all of 12 mice examined. In the ovariectomised females adrenal cortical hyperplasia occurred in 5 mice only, and a female type of glomerular capsule lining in 6 only. In only 1 mouse was there a change in sex character of the salivary glands. Nodules in the breast occurred in only 3·6 per cent of ovariectomised female mice, in comparison with 29 per cent in intact females. No evidence of post-castrational androgen was found in either sex.

### DISCUSSION

A lower incidence of hepatomas in breeding females compared with virgin females and males was confirmed in the C3Hf mice used in these experiments. This is in accordance with the findings of other authors working with this strain. Ovariectomy in females and castration in males had no effect on the incidence of hepatomas compared with that in intact animals. Although there was evidence of some post-castrational secretion of oestrogen in males, it did not affect the incidence of hepatoma. Administration of oestrogen to castrated males and females tended to lower the hepatoma incidence even when the dose was small, but this decrease did not beome statistically significant unless a fairly large dose of oestrone or a large dose of oestriol was given.

It is necessary in estimating the effects of treatment that survival ages must be comparable with normal because hepatoma arises late in life. In the C3Hf strain in these laboratories it was first seen at 16 months old, and only mice of 16 months and older are included in the results. Large amounts of oestrogens which significantly lowered hepatoma incidences, reduced survival rates also. Thus although treatment with 10 $\mu$g. oestrone and 200 $\mu$g. oestriol reduced the hepatoma incidence in ovariectomised virgin females to that of breeders it is possible that side effects such as bladder calculi with reduced survival rate and loss in weight may have been partly responsible for this. It is suggested that intermediate doses might be tested for an effect on hepatoma incidence.

### SUMMARY

1. The incidence of hepatoma in C3Hf breeding females was 6·8 per cent, that of intact virgin females 24·5 per cent, and that of ovariectomised virgin females 25 per cent.

2. Hepatoma incidence in C3Hf males was 29·8 per cent and in those castrated was 32 per cent.

3. Survival rates of ovariectomised mice given 5 $\mu$g. oestrone or 10 $\mu$g. of a mixture of oestrone, oestradiol and oestriol were similar to normal and ovariectomised virgin females. Five $\mu$g. oestrone given to castrated males did not reduce survival. Hepatoma incidences were 15·1, 12·2 and 12·5 per cent respectively.

4. Reduction in hepatoma incidence became statistically significant when larger amounts of oestrogens were given, but 10 $\mu$g. oestrone reduced survival and 200 $\mu$g. oestriol reduced weight and survival rate.

We wish to thank Dr. P. R. Peacock for his interest and advice and Dr. S. Iversen for help with statistics.

### REFERENCES

Agnew, L. R. C. and Gardner, W. U.—(1952) *Cancer Res.*, **12**, 757.

Andervont, H. B.—(1950) *J. nat. Cancer Inst.*, **11**, 581.

*Idem* and McEleney, W. J.—(1941) *Ibid.*, **1**, 737.

Brown, J. B.—(1956) *Lancet*, i, 704.

Burns, E. L. and Schenken, J. R.—(1940) *Amer. J. Cancer*, **39**, 25.—(1943) *Cancer Res.*, **3**, 691.

Gorer, P. A.—(1940) *Rep. Brit. Emp. Cancer Campgn.*, **17**, 232.—(1940) *J. Path. Bact.*, **50**, 17.

Heston, W. E. and Deringer, M. K.—(1953) *Proc. Soc. exp. Biol., N.Y.*, **82**, 731.
*Iidem* and Vlahakis, G.—(1960) *J. nat. Cancer Inst.*, **24**, 425.
Miller, E. W. and Pybus, F. C.—(1945) *Cancer Res.*, **5**, 84.
Parkes, A. S.—(1926) *Proc. roy. Soc.*, **100**, 151.
Pullinger, B. D.—(1947) *Brit. J. Cancer*, **1**, 177.—(1957) *Ibid.*, **11**, 249.—(1959) *Ibid.*, **13**, 99.—(1960*a*) *Ibid.*, **14**, 279.—(1960*b*) *Ibid.*, **14**, 502.—(1961) *Ibid.*, **15**, 127.
*Idem* and Iversen, S.—(1960) *Ibid.*, **14**, 267.
Pybus, F. C. and Miller, E. W.—(1942) *Rep. Brit. Emp. Cancer Campgn.*, **19**, 42.
Tannenbaum, A. and Silverstone, H.—(1949) *Cancer Res.*, **9**, 162, 724.